# Overcoming drug resistance of cancer cells by targeting the FGF1/FGFR1 axis with honokiol or FGF ligand trap

**DOI:** 10.3389/fphar.2024.1459820

**Published:** 2024-09-12

**Authors:** Jakub Szymczyk, Martyna Sochacka, Martyna Biadun, Katarzyna Dominika Sluzalska, Danuta Witkowska, Malgorzata Zakrzewska

**Affiliations:** ^1^ Department of Protein Engineering, Faculty of Biotechnology, University of Wroclaw, Wroclaw, Poland; ^2^ Department of Protein Biotechnology, Faculty of Biotechnology, University of Wroclaw, Wroclaw, Poland; ^3^ Institute of Health Sciences, University of Opole, Opole, Poland

**Keywords:** FGF1, FGFR1, drug resistance, honokiol, ligand trap, cancer, anti-cancer drugs, taltobulin

## Abstract

**Background:**

Chemoresistance of cancer cells, resulting from various mechanisms, is a significant obstacle to the effectiveness of modern cancer therapies. Targeting fibroblast growth factors (FGFs) and their receptors (FGFRs) is becoming crucial, as their high activity significantly contributes to cancer development and progression by driving cell proliferation and activating signaling pathways that enhance drug resistance.

**Methods:**

We investigated the potential of honokiol and FGF ligand trap in blocking the FGF1/FGFR1 axis to counteract drug resistance. Using PEAQ-ITC, we verified direct interaction of honokiol with the FGFR1 kinase domain. We then demonstrated the effect of FGF1/FGFR1 inhibition on taltobulin resistance in cells expressing FGFR1. Finally, we generated drug-resistant clones by prolonged exposure of cells with negligible FGFR levels to taltobulin alone, taltobulin and honokiol, or taltobulin and FGF ligand trap.

**Results:**

We demonstrated for the first time a direct interaction of honokiol with the FGFR1 kinase domain, resulting in inhibition of downstream signaling pathways. We revealed that both honokiol and FGF ligand trap prevent FGF1-dependent protection against taltobulin in cancer cells expressing FGFR1. In addition, we showed that cells obtained by long-term exposure to taltobulin are resistant to both taltobulin and other microtubule-targeting drugs, and exhibit elevated levels of FGFR1 and cyclin D. We also found that the presence of FGF-ligand trap prevents the development of long-term resistance to taltobulin.

**Conclusion:**

Our results shed light on how blocking the FGF1/FGFR1 axis by honokiol and FGF ligand trap could help develop more effective cancer therapies, potentially preventing the emergence of drug-resistant relapses.

## 1 Introduction

An important challenge for contemporary oncology is the emergence of resistance to drugs used in current anticancer therapies (Lu et al., 2022). Mechanisms driving chemoresistance include inhibition of apoptosis, drug neutralization, increased drug efflux, enhancement of DNA repair mechanisms, and mutations affecting drug-binding sites (Housman et al., 2014). An increasingly important role in the development of drug resistance has been attributed to growth factors and their receptors (Dai et al., 2004). Their intensified activity has been linked to reduced sensitivity to certain anti-cancer drugs, through stimulation of various metabolic processes, proliferation and motility of cancer cells (Turner and Grose, 2010; Guo et al., 2018; Szymczyk et al., 2021). Our previous studies indicated that activation of fibroblast growth factor (FGF)-induced signaling leads to increased resistance to microtubule-targeted drugs such as taltobulin, paclitaxel and vincristine (Szymczyk et al., 2022; Szymczyk et al., 2023). Moreover, clinical studies by others have demonstrated that selective inhibition of FGF receptor (FGFR)-dependent signaling pathways with specific inhibitors or ligand traps not only significantly impedes tumor growth, but also holds promise for preventing drug resistance in clinical practice among patients (Javle et al., 2018; Morgensztern et al., 2019; van Brummelen et al., 2020; Katoh et al., 2024). In light of these findings, further investigation of potential inhibitory effects on FGF/FGFR signaling may provide alternative therapeutic strategies.

Honokiol (HNK), a biphenolic compound extracted from the bark and foliage of *Magnoliaceae* plant species, has historically been used in traditional Chinese medicine to treat gastrointestinal disorders, coughs and allergic diseases (Banik et al., 2019). Extensive *in vitro* and *in vivo* studies have consistently highlighted honokiol’s remarkable efficacy against diverse cancer types, including lung, breast, skin, pancreatic, liver and prostate cancers, by targeting pathologically related pathways, such as MAPK, AKT, mTOR, NF-κB and STAT3 (Crane et al., 2009; Arora et al., 2011; Arora et al., 2012; Rajendran et al., 2012; Tian et al., 2013). Notably, in breast and lung cancer cells, honokiol has shown promising potential in overcoming drug resistance (Wang et al., 2017; Zang et al., 2020). Previous studies have indicated the ability of honokiol to inhibit epidermal growth factor receptor (EGFR) signaling through its direct interaction with the kinase domain of EGFR (Leeman-Neill et al., 2010; Song et al., 2016; Zang et al., 2020). To our knowledge, only one study has reported an inhibitory effect of honokiol on the FGF2/FGFR1 axis, but it did not address the aspect of drug resistance (Cen et al., 2018). This prompted us to investigate the inhibitory effects of honokiol on signaling cascades involved in the FGF1/FGFR1 axis. An alternative approach to inhibit FGFR activation involves the use of FGF ligand traps designed to target their natural ligands, mainly canonical (mitogenic) FGFs, present in the extracellular environment of the tumor (Presta et al., 2017; Taranto et al., 2024). These traps can range in structure from low-molecular-weight derivatives, e.g., steroid-based (NSC12), to complete protein subunits, such as the extracellular domain of FGFR1 (FP-1039) (Tolcher et al., 2016; Taranto et al., 2024). Mimicking the natural structure of the receptor, the FGF ligand trap competes with the receptors on the cell surface for binding to the ligand, effectively blocking the activation of signaling cascades triggered by FGF proteins (Presta et al., 2017). This strategy is also being investigated in clinical trials, e.g., in the treatment of lung cancer in combination with paclitaxel (Morgensztern et al., 2019). Our study aimed to verify whether blocking FGF1/FGFR1 activity with honokiol or an FGF ligand trap is able to re-sensitize cancer cells to taltobulin and prevent the development of long-term drug resistance.

## 2 Materials and methods

### 2.1 Antibody and reagents

Primary antibodies including anti-FGFR1 (FGFR1) (#9740), anti-phospho-PLCγ1 (Tyr783) (p-PLCγ) (#14008), anti-phospho-AKT (Ser473) (p-AKT) (#9271), anti-phospho-p44/42 (Thr202/Tyr204) MAP kinase (p-ERK1/2) (#9101), and anti-Cyclin D1 (#2978) were from Cell Signaling Technology (Danvers, MA, United States); anti-phospho-FGFR (Tyr653/Tyr654) (p-FGFR) (#06-1433) and anti-γ-tubulin (γ-tubulin) (#T6557) were obtained from Sigma Aldrich (St Louis, MO, United States). Horseradish peroxidase-conjugated secondary anti-mouse and anti-rabbit antibodies were from Jackson Immuno-Research Laboratories (Cambridge, United Kingdom). Geneticin (G-418) was from BioShop (Puck, Poland) and Penicillin-Streptomycin Solution (Pen/Strep) was from Biowest (Nuaille, France). Fetal bovine serum (FBS) was from Thermo Fisher Scientific (Waltham, MA, United States). Heparin was from Sigma-Aldrich.

### 2.2 Anticancer drugs

Honokiol and paclitaxel were purchased from Sigma-Aldrich. Taltobulin (HTI-286) was from MedChem Express (Monmouth Junction, NJ, United States). Vincristine and BGJ 398, were from Selleckchem (Houston, TX, United States).

### 2.3 Plasmids

The pCDFDuet-1 plasmid containing genes for FGFR1_KD and PTP1B (phosphotyrosine phosphatase 1B) proteins was constructed based on previous work (Yosaatmadja et al., 2015) and obtained from Gene Universal (Newark, NJ, United States).

### 2.4 Recombinant proteins

Recombinant proteins: human FGF1 and FGF ligand trap (extracellular domain of FGFR1c fused with Fc region, ECD_FGFR1-Fc) were produced as previously described (Zakrzewska et al., 2004; Sokolowska-Wedzina et al., 2014). The FGFR1 kinase domain (FGFR1_KD) was expressed in *E. coli BL21 (DE3)* following established protocols (Yosaatmadja et al., 2015). PTP1B was co-expressed to facilitate complete dephosphorylation of FGFR1_KD, ensuring a homogeneous sample for subsequent analysis. Briefly, *E. coli* bacteria were transformed with the pCDFDuet-1 plasmid carrying FGFR1_KD and cultured in Terrific Broth medium supplemented with 50 mg/mL ampicillin at 37°C until the OD_600_ reached 1.0. Protein expression was induced by adding 1 mM IPTG, lowering the culture temperature to 18°C. After 18 h of culture, bacteria were harvested by centrifugation at 5,000 × g and resuspended in Ni buffer A (20 mM Tris-HCl, 10 mM imidazole, 300 mM NaCl, 2 mM TCEP with protease inhibitors (cOmplete EDTA-free protease inhibitor, Roche, Indianapolis, IN, United States), pH 7.8), and then centrifuged at 20,000 × g for 30 min at 4°C. Protein purification involved applying the supernatant to a 5 mL His-Trap column (GE Healthcare, Chicago, IL, United States) pre-equilibrated with Ni buffer A, followed by elution with a linear gradient of Ni buffer B (20 mM Tris-HCl, 500 mM imidazole, 300 mM NaCl, 2 mM TCEP, pH 7.8). Protein-containing fractions were pooled and desalted using a HiTrap desalting column (GE Healthcare) into Tris buffer (20 mM Tris-HCl, 150 mM NaCl, 2 mM TCEP, pH 7.4). Protein purity was confirmed by SDS-PAGE.

### 2.5 Cell lines

The U2OS (human osteosarcoma) and DMS114 (human small cell lung cancer) cell lines were obtained from the American Type Culture Collection (ATCC, Manassas, VA, United States). The U2OS cells stably transfected with pcDNA3.1 vector encoding full-length FGFR1c (U2OSR1) were generated as previously described (Poźniak et al., 2021). For U2OS cells, culture conditions included DMEM (Biowest) supplemented with FBS and antibiotics (Pen/Strep). For U2OSR1 cells, the addition of 1 mg/mL geneticin was used. DMS114 cells were maintained in Waymouth’s MB 752/1 medium (ATCC) supplemented with 10% FBS and antibiotics (Pen/Strep). All cell lines were incubated at 37°C in an atmosphere of 5% CO_2_.

### 2.6 Analysis of signaling pathways

To assess the effect of honokiol on FGFR1 and its downstream signaling pathways, U2OSR1 cells were incubated for 6 h without serum and then were treated with 30 µM honokiol or its solvent, 0.1% DMSO, as a control for 10 min before FGF1 stimulation. Next, cells were treated with 10 ng/mL of FGF1 in the presence of heparin for the specified time (0, 5, 15, 60 min). The cells were then lysed with sample buffer and sonicated, followed by SDS-PAGE and Western blotting using specific primary antibodies recognizing the phosphorylated forms of the signaling proteins. Membranes were incubated with HRP-conjugated secondary antibodies and protein bands were visualized using a chemiluminescent substrate on ChemiDoc station (BioRad, Hercules, CA, United States).

### 2.7 Isothermal microcalorimetry

Interactions between honokiol and the FGFR1 kinase domain (FGFR1_KD) were measured by isothermal microcalorimetry (Malwern PEAQ-ITC) in buffer containing 20 mM Tris-HCl, 150 mM NaCl, 2 mM TCEP, 0.1% DMSO, pH 7.4. After stabilizing the device at 25°C, 40 µL of a honokiol (0.5 mM) was used to titrate 200 µL of a protein (23 µM). Each titration consisted of 19 consecutive injections with a 150 s interval between each aliquot and a stirring speed of 750 rpm. The heat of a dilution from the corresponding control titration was subtracted before data fitting. The initial injection of 0.4 µL was removed from each data set to eliminate the effect of titrant diffusion through the syringe tip during the equilibration process. Data were processed using MicroCal PEAQ-ITC Analysis Software.

### 2.8 Cell viability assay

Cancer cells were cultured in the appropriate medium in 96-well plates (at the specified densities: 1 × 10^4^ cells/well for U2OS and U2OSR1 and 4 × 10^4^ for DMS114) and treated with various substances such as 30 µM honokiol, 10 μg/mL ECD_FGFR1-Fc (FGF ligand trap), 1 µM BGJ 398 (FGFR inhibitor), 5 nM taltobulin, 20 nM paclitaxel or 10 nM vincristine (tubulin disrupting drugs) in the presence or absence of 10 ng/mL FGF1 and with heparin (10 U/mL). 0.1% DMSO was used as a control for each drug. After a 48-h incubation at 37°C, the cell viability was determined using PrestoBlue Cell Viability Reagent (Thermo Fisher Scientific). The fluorescence emitted by the reduced form of the dye was quantified at 590 nm after excitation at 560 nm, using a Tecan Infinite M1000 PRO plate reader. Results were normalized to untreated cells or to cells treated with inhibitors alone.

### 2.9 Development of drug-resistant cancer cell lines

Drug-resistant cancer cell lines were derived from U2OS cells maintained in DMEM medium supplemented with 10% FBS, Pen/Strep, and exposed to 5 nM taltobulin alone (U2OS_TR), or 5 nM taltobulin and 30 μM honokiol (U2OS_TR_HNK), or 5 nM taltobulin and 10 μg/mL FGF ligand trap (U2OS_TR_LT) over 4 cycles, each lasting 1 week. During each cycle, cells were subjected to a 4-day drug exposure followed by a 3-day drug-free interval. Cells that survived the extended drug exposure were isolated and cultured for an additional period of approximately 30 days without drugs to promote cell proliferation.

### 2.10 Bright-field microscopy

U2OS, U2OS_TR, U2OS_TR_HNK and U2OS_TR_LT cells were plated at a density of 1 × 10^4^ cells/well in DMEM with 10% FBS and Pen/Strep. Cells were then washed with PBS and fixed by adding 4% paraformaldehyde (PFA) for 15 min at room temperature. Bright-field microscopy was performed using a Zeiss Axio Observer Z1 microscope (Zeiss, Oberkochen, Germany) with an A-Plan Objective 10x/0.25 M27 objective and an Axiocam 503 camera. Image analysis was performed using ZEN 2.3 software (Zeiss).

### 2.11 Cell migration assay

Cell migration was analyzed using the IncuCyte^®^ Cell Migration and Invasion System (Essen BioScience, Royston, United Kingdom). Parental U2OS cells and their derived cell lines (U2OS_TR, U2OS_TR_HNK, and U2OS_TR_LT) were seeded into a 96-well IncuCyte^®^ ImageLock plate at a density of 4.5 × 10^4^ cells/well, in DMEM with 10% FBS and Pen/Strep and scratched with IncuCyte^®^ WoundMaker. Cells were then treated with 5 nM TLT. 0.1% DMSO was used as a control for taltobulin. Wound density was monitored over 30 h, with images automatically acquired every 2 h using an IncuCyte^®^ ZOOM 10× objective, and then analyzed within the wound area using the IncuCyte^®^ ZOOM GUI Version: 2018A Software package. Relative wound density was measured after 20 h of drug treatment.

### 2.12 Statistical analysis

Statistical analysis was performed using an unpaired two-tailed t-test with GraphPad Prism 5 software (GraphPad Software, CA, United States) to determine whether there was a significant difference between the means of two independent groups. Data are expressed as the means ± standard deviation (SD) or ± standard error of the mean (SEM) obtained from at least three independent experiments, each consisting of three replicates. The SD was used to illustrate variability within a single cell line when different doses of inhibitors were used, while the SEM was used to show differences between different cell lines in response to the drugs. The significance of the results was categorized using the following notations: * for *p* ≤ 0.05, ** for *p* ≤ 0.01, *** for *p* ≤ 0.001. Results that did not reach statistical significance, with a *p*-value greater than 0.05, were marked as “ns” (not significant).

## 3 Results

### 3.1 Honokiol inhibits FGF-induced cell signaling through direct interaction with the kinase domain of FGFR1

**FIGURE 1:**
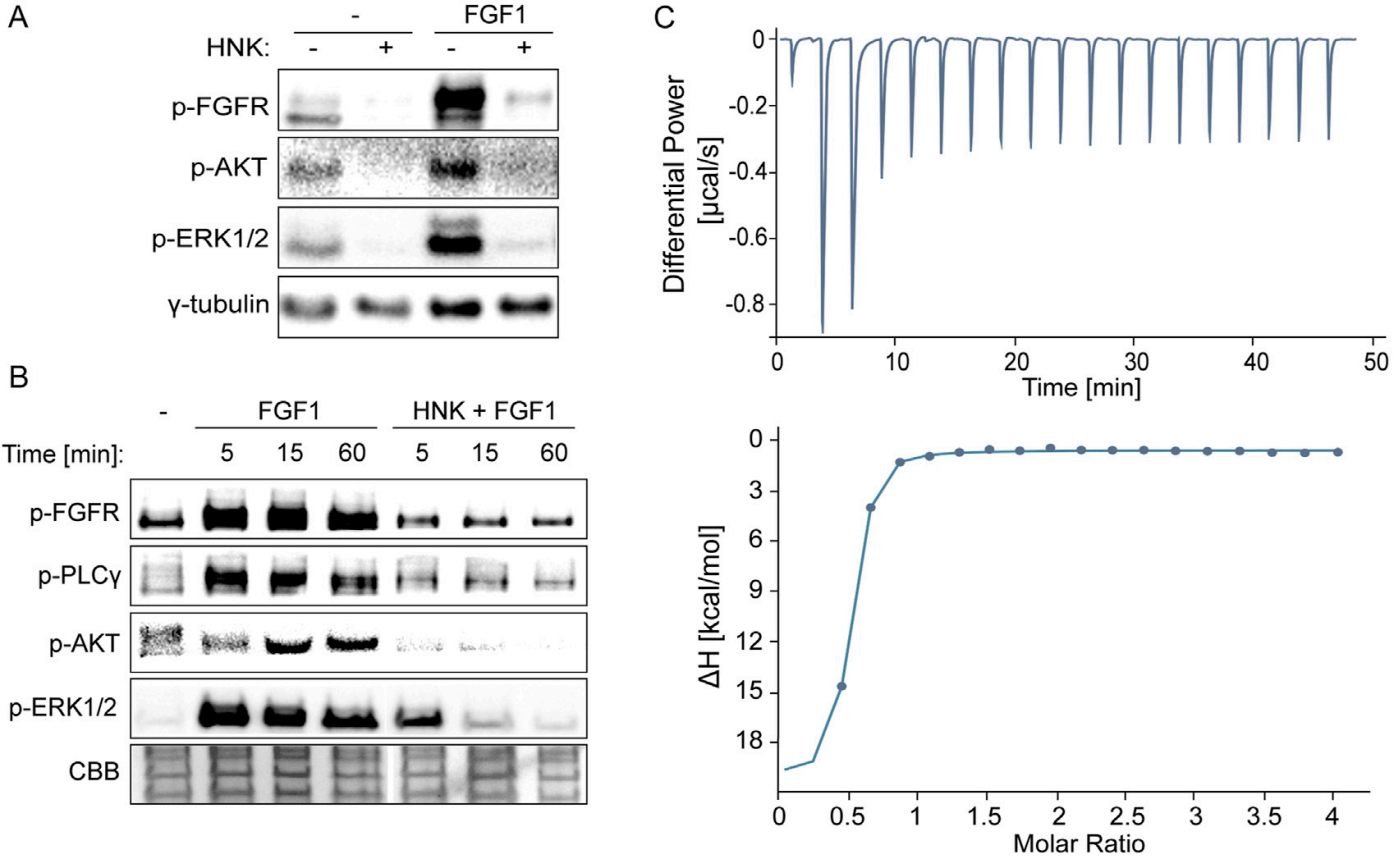
Effect of honokiol on inhibition of FGFR-dependent signaling through direct interaction with the FGF receptor 1 kinase domain (FGFR1_KD). **(A, B)** Western blotting analysis demonstrating inhibition of FGFR1 activation and downstream cell signaling. U2OSR1 cells were serum-starved for 6 h, then treated with 30 µM honokiol for 15 min, and finally stimulated with 10 ng/mL FGF1 for 15 min **(A)** or 5, 15, and 60 min **(B)**. Cells were then lysed using sample buffer and sonicated, followed by SDS-PAGE and Western blotting. Levels of phosphorylated signaling proteins, FGFR, AKT, ERK1/2, and PLCγ were analyzed using specific antibodies. Anti-γ-tubulin antibodies or Coomassie Brilliant Blue (CBB) protein staining were used as loading controls. **(C)** Binding between the FGFR1_KD and honokiol measured by PEAQ-ITC. 0.5 mM honokiol was used to titrate 23 µM FGFR1_KD. Each titration consisted of 19 consecutive injections with an interval of 150 s between each aliquot and a stirring speed of 750 rpm. Data were processed using MicroCal PEAQ-ITC Analysis Software, allowing calculation of binding parameters: Kd = 127 ± 64.1 nM, N = 0.474 ± 0.018 (ratio 2:1).

Here, we examined the effect of honokiol on FGFR1 activity, building on previous work demonstrating its role in EGFR inhibition (Song et al., 2016). Initially, we verified FGFR1 phosphorylation and signaling pathway activation in FGF1-stimulated U2OS cells overexpressing FGFR1 (U2OSR1) in the presence of honokiol. Cells incubated for 6 h without serum to eliminate external activating factors were treated with 30 µM honokiol for 10 min before 15-min FGF1 stimulation in the presence of heparin. The concentration of honokiol was chosen based on previous studies (Pan et al., 2017; Pearson et al., 2018), but also to achieve both complete inhibition of FGFR1-dependent cell signaling and a cytotoxic effect on U2OSR1. In cells treated with FGF1 alone, a strong signal of activated FGFR and downstream signaling manifested by phosphorylation of AKT and ERKs was observed. However, in cells treated with honokiol, FGF1 did not induce activation of either FGFR or AKT and ERKs (Figure 1A).

Moreover, our study of the kinetics of signaling activation at 5, 15, and 60 min after FGF1 stimulation showed consistent, long-term prevention by honokiol of FGFR-dependent signaling activation involving ERKs, AKT and PLCγ (Figure 1B).

Based on these observations, and taking into account honokiol’s interaction with the EGFR kinase domain, we verified the possibility of its direct interaction with the FGFR1 tyrosine kinase. Using micro-ITC measurements, we detected rapid, strong binding with strength in the nanomolar range (Kd = 127 ± 64.1 nM) in a 2:1 ratio (FGFR1_KD to HNK) (Figure 1C). It should be noted, however, that such a strong affinity may suggest that the binding parameters are not precisely determined due to the limitations of the ITC technique (Turnbull and Daranas, 2003). Nevertheless, it is clear that this interaction is enthalpy-driven, and the unfavorable entropy contribution is much lower (Supplementary Figure S1).

### 3.2 Honokiol prevents protective effects of FGF1 in taltobulin-treated cells expressing FGFR1

**FIGURE 2:**
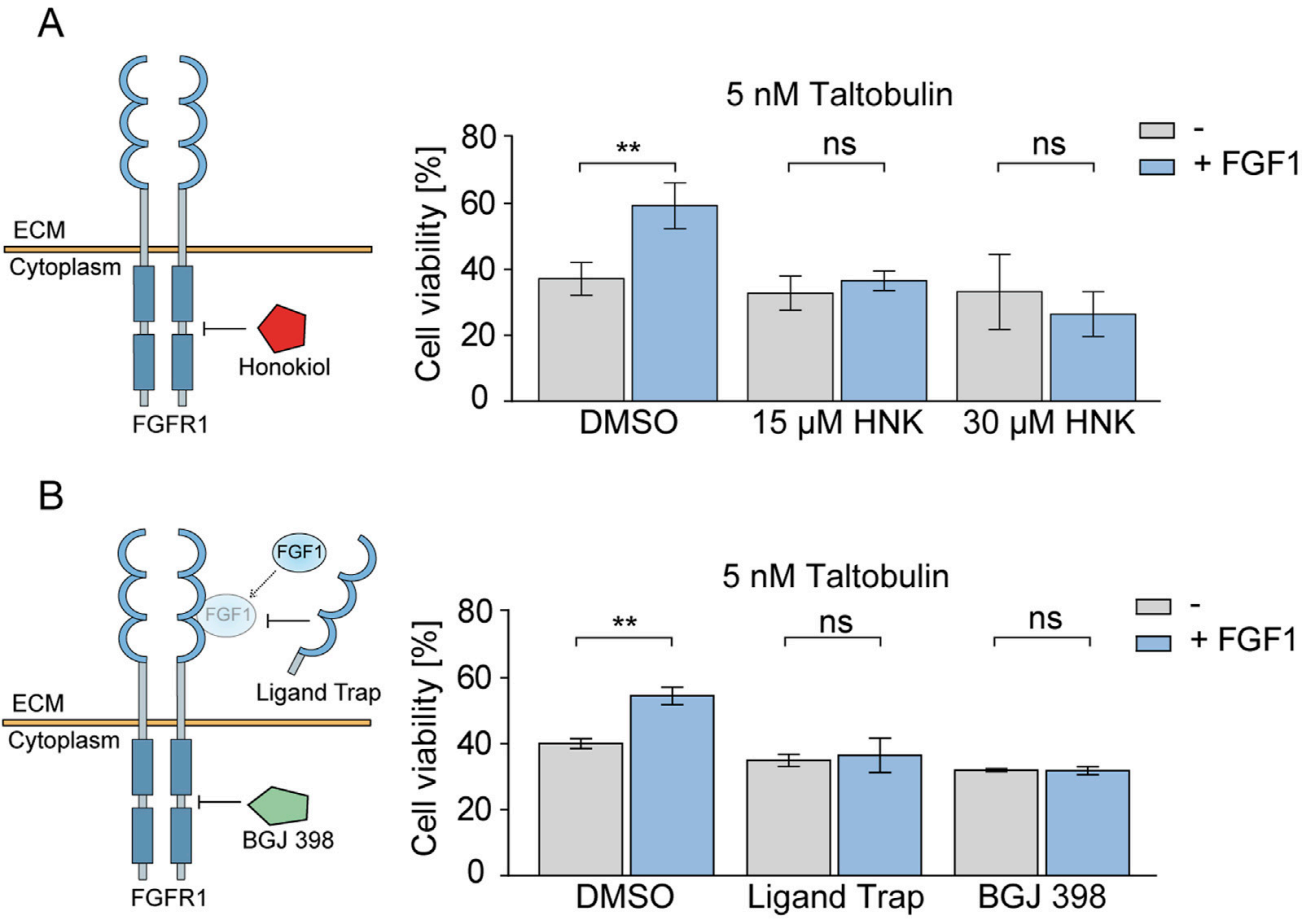
Effect of honokiol and ligand trap on the FGF1-induced protection against taltobulin via FGFR1 inhibition. **(A)** Effect of honokiol (15 μM or 30 μM) on the viability of U2OSR1 cells treated with 5 nM taltobulin for 48 h in the presence or absence of 10 ng/mL FGF1 and 10 U/mL heparin. **(B)** Effect of 10 μg/mL FGF ligand trap (ECD_FGFR1-Fc) on the viability of U2OSR1 cells treated with 5 nM taltobulin for 48 h in the presence or absence of 10 ng/mL FGF1 and 10 U/mL heparin. 1 μM BGJ 398, a potent FGFR tyrosine kinase inhibitor, was used as a control. Cell viability was assessed using the PrestoBlue assay. All data were normalized to untreated cells. Statistical analysis was performed using an unpaired two-tailed t-test with GraphPad Prism 5. Data are shown as means ± SD from three independent experiments (n = 3) with three replicates each. Statistical significance was defined as: **p ≤ 0.01, no significant differences (*p* > 0.05) indicated as “ns”.

Our previous studies have unequivocally shown that FGF1 protects U2OSR1 cells, DMS114 cells and MCF7 cells against the effects of cytotoxic drugs such as taltobulin (Szymczyk et al., 2022; Szymczyk et al., 2023). Therefore, having confirmed the inhibitory effect of honokiol on FGFR1 activity, we focused on determining whether honokiol could attenuate or abolish the protective activity of FGF1 in cancer cells exposed to taltobulin. To this end, U2OSR1 cells were treated with the drug and FGF1 in the absence or presence of honokiol (at concentrations of 15 μM and 30 µM) for 48 h, and then their viability was assessed. These two concentrations of honokiol, 15 µM and 30 μM, were chosen to verify which concentration more effectively inhibits FGF1-dependent protection against taltobulin in both tested cell lines. In subsequent experiments, a higher concentration of 30 µM was used, due to the fact that in DMS114 cells the effect was statistically significant only for 30 µM. As expected, in cells untreated with honokiol, FGF1 stimulation results in decreased cell sensitivity to taltobulin, but in the presence of honokiol, the protective effect of FGF1 was abolished (Figure 2A). A similar effect was observed in DMS114 lung cancer cells, also overproducing FGFR1 (Supplementary Figure S2).

In addition, we investigated to what extent the inhibitory effect on FGFR1 activation observed with honokiol is comparable to that of FGF ligand trap. We used the recombinant extracellular domain of FGFR1 fused to an Fc (Ligand Trap) fragment to eliminate the effect of FGF1 on surface FGF receptors through its competitive binding (Harding et al., 2013). As a control, we used a well-known FGFR inhibitor, BGJ 398, which is currently in clinical trials (Javle et al., 2018; Lassman et al., 2022). In both cases, the protective effect of FGF1 was abolished in U2OSR1 cells (Figure 2B).

### 3.3 FGF ligand trap prevents long-term resistance to taltobulin

**FIGURE 3:**
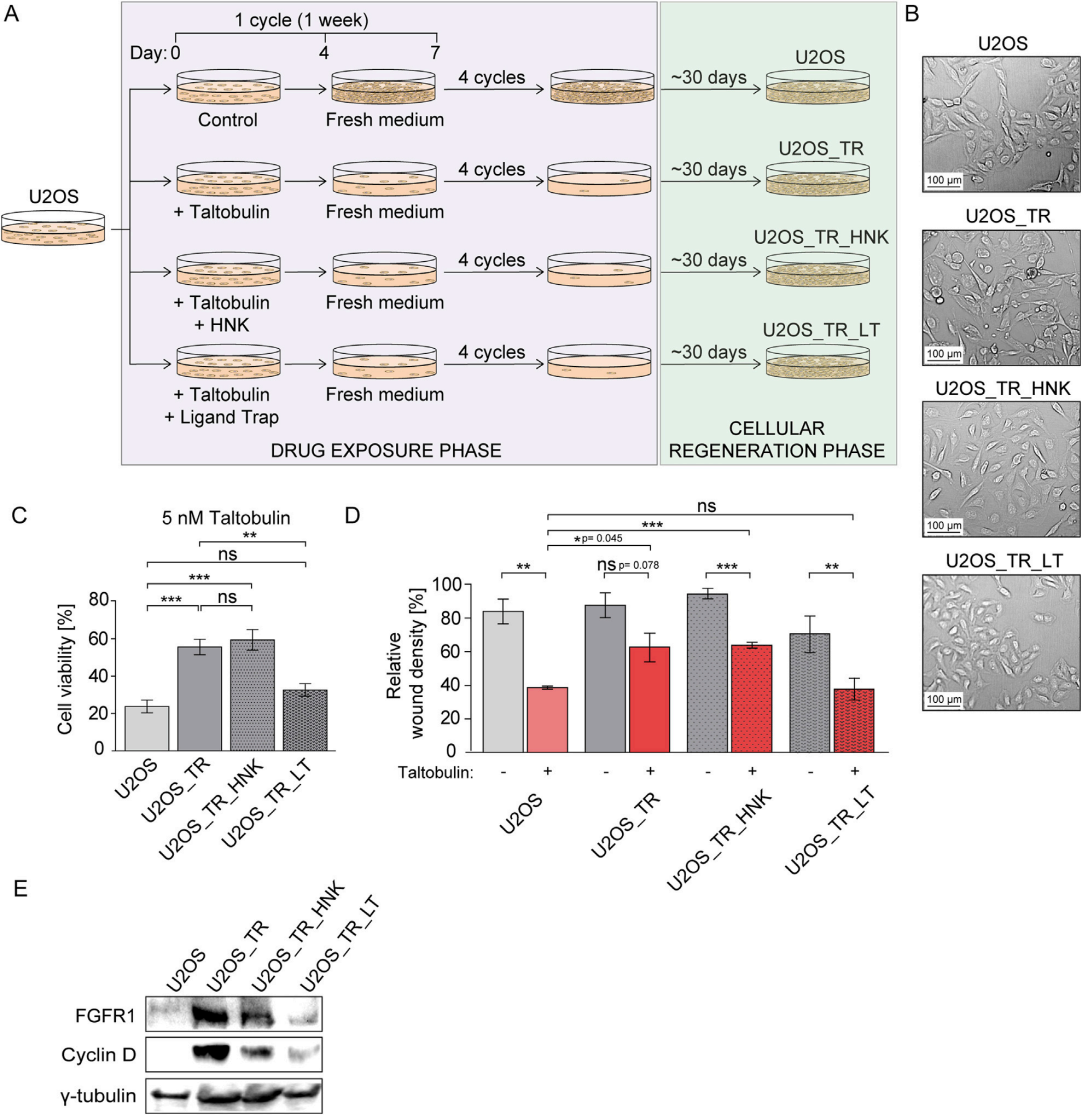
Effect of honokiol and ligand trap on the development of drug resistance in U2OS cells long-term treated with taltobulin. **(A)** Schematic illustrating the process of obtaining taltobulin-resistant cells. U2OS cells were treated with taltobulin alone or in the presence of honokiol (HNK) or a ligand trap (LT) for 4 weeks in weekly cycles: 4 days with 5 nM taltobulin (drug exposure phase) and 3 days without the drug (cellular regeneration phase). After 4 cycles, cells were then cultured for about 30 days to promote the growth of cells remaining after drug exposure. **(B)** Images of parental U2OS cells and their derived cell lines (U2OS_TR, U2OS_TR_HNK, and U2OS_TR_LT) after the drug exposure phase and cellular regeneration phase captured using bright-field microscopy at ×10 magnification. **(C)** Evaluation of long-term exposure of U2OS cells to taltobulin on the development of chemoresistance. Parental U2OS cells and derived U2OS_TR, U2OS_TR_HNK and U2OS_TR_LT were treated with 5 nM taltobulin for 48 h, and then their viability was assessed using the PrestoBlue assay. Data were normalized to untreated cells. **(D)** Effect of taltobulin on the migratory capacity of tested taltobulin-resistant cell lines. U2OS cells and their derivatives were seeded onto ImageLock 96 well plates and scratched with IncuCyte^®^ WoundMaker. The cells were then treated with 5 nM taltobulin. The rate of scratch closure was monitored for 30 h using the IncuCyte^®^ Cell Migration and Invasion System (data cut-off: 20 h). Relative wound density was normalized by taking into account both the density of cells in the wound area and the width of the wound. Data are presented as a percentage representing the wound area occupied by migrating cells over time. Statistical analysis was performed using an unpaired two-tailed t-test with GraphPad Prism 5. All data are presented as means ± SEM from three independent experiments (n = 3) with three replicates each. Statistical significance was defined as: *p ≤ 0.05, **p ≤ 0.01, ***p ≤ 0.001, no significant differences (*p* > 0.05) indicated as “ns”. In addition, exact *p*-values ranging from 0.04–0.08 are given. **(E)** Western blot analysis of FGFR1 and cyclin D levels in taltobulin-resistant U2OS cell lines. Cells were lysed using sample buffer and sonicated, followed by SDS-PAGE and Western blotting. Levels of proteins, FGFR1 and Cyclin D were analyzed using specific antibodies. Anti-γ-tubulin antibody was used as a loading control.

Next, we wanted to test whether inhibition of FGFR1 activity by honokiol and FGF ligand trap in cancer cells with low levels of FGF receptors could reverse the acquisition of long-term multidrug resistance. To achieve this, we selected human osteosarcoma cells (U2OS) characterized by minimal FGFR levels (Świderska et al., 2018). Parental U2OS cells were treated with taltobulin (5 nM) alone or in combination with 30 μM honokiol or 10 μg/mL FGF ligand trap (similar to previous studies (Blackwell et al., 2016)) for 4 weeks. This was followed by a four-week period of recovery of these cells (Figure 3A).

Our preliminary observations revealed morphological changes in cells treated with taltobulin alone or with taltobulin and honokiol, i.e., a transition from oval to elongated cells, which was not observed in cells treated with taltobulin in combination with the ligand trap (Figure 3B). The U2OS_TR (U2OS cultured in the presence of taltobulin), U2OS_TR_HNK (U2OS cultured in the presence of taltobulin and honokiol) and U2OS_TR_LT (U2OS cultured in the presence of taltobulin and ligand trap) lines were then evaluated for acquired resistance to taltobulin. The cells were treated with 5 nM taltobulin for 48 h, after which their viability was examined and compared to parental U2OS cells (Figure 3C). We found that U2OS_TR cells exhibited 35.2% (±2.8%) lower sensitivity to taltobulin relative to U2OS cells. Similarly, U2OS_TR_HNK cells showed a 39% (±6.5%) decrease in sensitivity, indicating that the presence of honokiol did not prevent the development of taltobulin resistance. It is worth noting that U2OS_TR_LT cells showed taltobulin sensitivity comparable to the parental cells (24% ± 3.4% for U2OS vs. 33% ± 3.8% for U2OS_TR_LT). Thus, our results indicate that FGF ligand trap, unlike honokiol, can prevent the acquisition of long-term resistance when administered concomitantly with the drug in cells with low level of FGFRs.

We then wanted to investigate whether U2OS_TR cells had developed resistance to other drugs with similar mechanisms of action, and whether the ligand trap could prevent this resistance. Cells were treated with 20 nM paclitaxel or 10 nM vincristine for 48 h (Szymczyk et al., 2022), and then their viability was assessed (Supplementary Figure S3). U2OS_TR cells developed resistance to both alternative drugs, showing reduced sensitivity to their toxicity compared to parental cells.

The development of drug resistance observed in cancer cells can be caused by various factors. One of them is epithelial-mesenchymal transition (EMT) during which cells often change their morphology from round to elongated (Singh et al., 2018), which we observed in U2OS_TR and U2OS_TR_HNK cells, while we did not observe this in U2OS_TR_LT cells, which did not develop resistance to taltobulin. We then examined the ability of these cells to migrate in the presence of the drug, assessing their ability to invade the wound. Cells cultured under optimal conditions were scratched using IncuCyte^®^ WoundMaker, and then their medium was exchanged for one containing 5 nM taltobulin. Cells were observed for 30 h, and wound closure images were taken every 2 h. The most significant differences were observed after 20 h (Figure 3D). Parental U2OS cells exhibited a significantly slower rate of wound closure in the presence of taltobulin. For U2OS_TR cells treated with the drug, the rate of wound closure rate was similar to untreated cells. The results confirm that U2OS_TR cells are less sensitive to taltobulin than U2OS cells, and that re-exposure to taltobulin no longer affects the migration of these cells. As in the previous experiment, U2OS_TR_LT cells were sensitive to taltobulin, i.e., the rate of wound closure was the same as that of the parental cells, confirming that the ligand trap prevented the acquisition of resistance to this drug. An interesting effect was observed in U2OS_TR_HNK cells, in which the rate of wound closure when treated with taltobulin was slower than in its absence, but still higher than in the case of parental cells. This result, different from the effects observed in viability analyses, raises questions about the effect of honokiol on the development of long-term resistance in U2OS cells.

Finally, we also evaluated the levels of FGFR1 and cyclin D in parental U2OS cells and all generated drug-resistant cell lines (Figure 3E). Interestingly, taltobulin exposure caused a significant increase in FGFR1 expression in the U2OS_TR line compared to the parental U2OS cell line, where FGFR1 levels were very low. U2OS_TR_HNK cells also showed increased levels of FGFR1, albeit lower than in U2OS_TR cells. Importantly, U2OS_TR_LT cells that had not acquired taltobulin resistance did not show increased FGFR1 expression. Another noteworthy phenomenon was the appearance of high levels of cyclin D in drug-resistant cells (U2OS_TR and U2OS_TR_HNK), the amount of which was below detection levels in parental cells. In cells cultured with ligand trap (U2OS_TR_LT) without acquired drug-resistance, cyclin D levels were very low.

## 4 Discussion

Understanding the mechanism of action of growth factors and their receptors appears to be crucial in designing effective anti-cancer therapies. In recent years, more and more attention has been paid to FGF and FGFR proteins in the context of their role in the emergence of drug resistance and the possibility of developing more effective targeted therapies (Turner and Grose, 2010; Szymczyk et al., 2021; Ornitz and Itoh, 2022; Katoh et al., 2024). Since others have suggested an effect of honokiol on EGFR kinase activity (Leeman-Neill et al., 2010; Cen et al., 2018), in this study we set out to investigate how honokiol affects FGFR-dependent signaling and the development of drug resistance in cells expressing FGFR1. Our goal was to validate its potential for use as a new therapeutic strategy or as an adjunct to existing therapies. In addition, we used an FGF ligand trap based on the extracellular domain of FGFR1 fused to an Fc fragment to compare its efficacy to that of honokiol in combating drug resistance.

We observed blocking of FGFR1 and downstream signaling proteins phosphorylation in honokiol-treated U2OSR1 cells. These findings suggest that honokiol has an inhibitory effect on the activation of FGFR-dependent pathways, similar to EGFR. To test whether this action is due to direct interaction of honokiol with FGFR1, we produced a recombinant kinase domain in the *E. coli* system and used it to analyze honokiol binding by applying the ITC technique.

We showed that honokiol binds to FGFR1 kinase and blocks downstream signaling, acting in a similar manner to well-established FGFR inhibitors (e.g., BGJ 398) (Guagnano et al., 2011). In the next step, we investigated whether honokiol could attenuate or nullify the protective effects of FGF1 on FGFR1-expressing cancer cells treated with the drug targeting tubulin polymerization, taltobulin. We demonstrated that honokiol effectively abrogated the protective effect of FGF1 in both U2OSR1 and DMS114 cells, increasing their sensitivity to the cytotoxic properties of taltobulin. A similar response was observed with the low-molecular-weight inhibitor BGJ 398, which, when added to a paclitaxel/carboplatin regimen, increased the cytotoxic effect of these drugs in ovarian cancer cells (Cha et al., 2017). To our knowledge, FGF ligand trap activity has not yet been directly linked to overcoming FGFR-dependent drug resistance. However, a novel FGF ligand trap, NSC12, has shown suppressive effect on bortezomib-resistant multiple myeloma cells (Taranto et al., 2024). Both FGF ligand traps and the FGFR inhibitors are currently undergoing clinical trials for their efficacy in anticancer therapies (Javle et al., 2018; Morgensztern et al., 2019; van Brummelen et al., 2020). These results point to the potential therapeutic benefits of the ligand trap (extracellular domain of FGFR1c) and honokiol in counteracting drug resistance, particularly in scenarios where FGF1-mediated protection is involved.

In our study, we also derived several taltobulin-resistant U2OS cell lines following long-term exposure to the drug alone or in combination with honokiol or FGF ligand trap. The generated U2OS_TR line (obtained by treatment with taltobulin alone) exhibited reduced sensitivity to re-exposure to taltobulin as well as other drugs with a similar mechanism of action, such as paclitaxel and vincristine. Furthermore, U2OS_TR cells have demonstrated increased migration in the presence of taltobulin compared to parental cells. Interestingly, we also found that U2OS_TR cells had increased expression of FGFR1 and cyclin D, while the levels of these proteins in the parental cells were very low (Alao et al., 2006; Świderska et al., 2018). Both proteins are associated with the phenomenon of epithelial-mesenchymal transition (EMT), which is also associated with drug resistance of cancer cells (Kurimoto et al., 2016; Shee et al., 2018). This indicates that long-term exposure to a microtubule-targeting drug significantly increases FGFR1 expression levels, which appears to be crucial for cell survival. The presence of a ligand trap in the process of long-term exposure to taltobulin led to obtaining of a cell line that was not resistant to taltobulin after the regeneration phase (30 days).

Undoubtedly, honokiol prevents the development of drug resistance induced by FGF1 in cells overexpressing FGFR1. Nevertheless, its effect on inhibiting long-term drug resistance in U2OS cells is unclear. The U2OS cells treated with taltobulin in the presence of honokiol (U2OS_TR_HNK) still manifested lower sensitivity to taltobulin in the proliferation assay compared to the parental cell, as did resistant clones (U2OS_TR). However, the migration pattern in the presence of the drug differed between U2OS_TR and U2OS_TR_HNK cells. This may be due to several factors, such as the rapid degradation of honokiol in the medium, as a result of which the activity of FGFR1, expressed at significantly increased levels in response to taltobulin, is not continuously inhibited. In contrast, the increased effectiveness of a stable ligand trap in counteracting resistance may be due to blocking FGF proteins present in the medium, and therefore blocking FGFR1 expression, at a higher level of effectiveness.

Despite the promising results, further studies are needed to gain a deeper understanding of the mechanisms involved in the process of counteracting resistance by honokiol and the trap ligand. It is essential to investigate the properties and stability of honokiol in order to assess its potential as an adjunctive therapy for cancer treatment. *In vivo* studies using mouse models or 3D cultures should be conducted to confirm the efficacy of honokiol and the ligand trap in systems more analogous to human physiology.

## 5 Conclusion

Our study emphasizes the potential of honokiol treatment as an effective supporting therapy for overcoming drug resistance in cancer treatment. By targeting FGFR1 kinase, honokiol appears to be a promising compound for enhancing the efficacy of anti-cancer drugs. However, its ability to prevent the development of long-term drug resistance in cells with low levels of FGFR expression remains questionable, emphasizing the complex interactions between honokiol, FGF signaling and resistance mechanisms. Nevertheless, we believe that our results, demonstrating the effects of honokiol and FGF ligand trap, provide a basis for further research and clinical trials on new alternative strategies to combat drug resistance.

## Data Availability

The original contributions presented in the study are included in the article/Supplementary Material, further inquiries can be directed to the corresponding author.
