# Supplementary material for: Overcoming drug resistance of cancer cells by targeting the FGF1/FGFR1 axis with honokiol or FGF ligand trap
Source: Front Pharmacol. 2024 Sep 12;15:1459820. doi: 10.3389/fphar.2024.1459820 (PMC11424896; doi:10.3389/fphar.2024.1459820)
Supplement: Supplementary file 1 [file DataSheet1.pdf]

10.3389/fphar.2024.1459820

SUPPLEMENTARY DATA

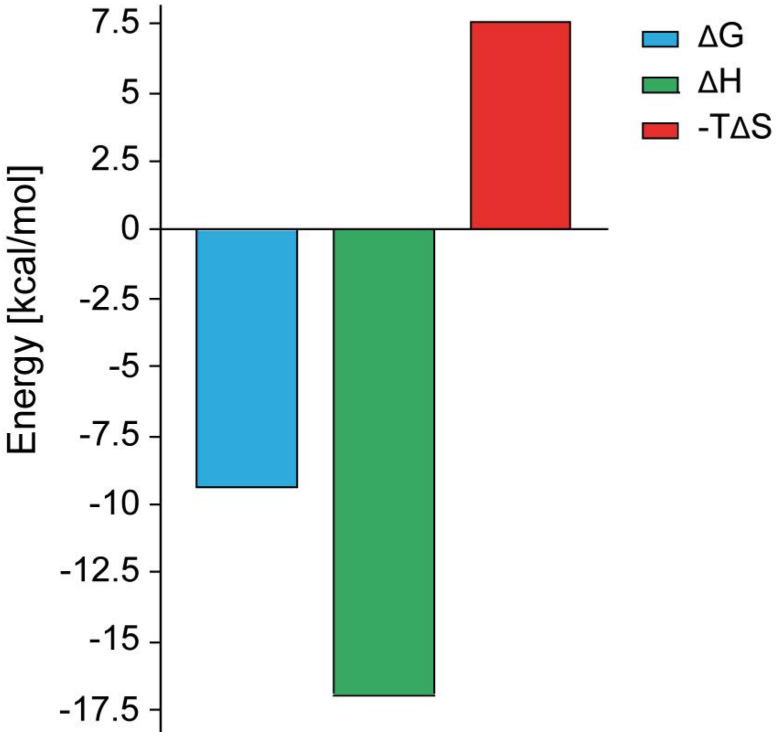

Supplementary figure 1. Thermodynamic parameters of the interaction between FGFR1_KD and honokiol measured by PEAQ-ITC: ΔG (Gibbs free energy), ΔH (enthalpy), and -TΔS (entropy change).

10.3389/fphar.2024.1459820

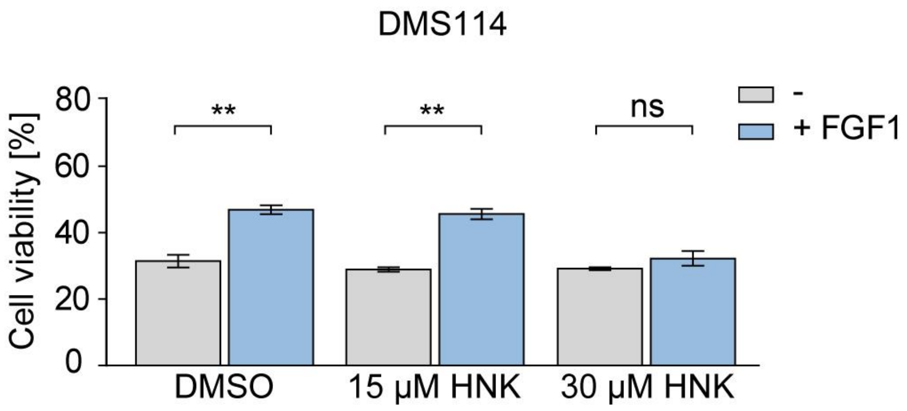

Supplementary figure 2. Effect of honokiol on the FGF1-induced protection against taltobulin *via* FGFR1 inhibition. DMS114 cells were treated with 5 nM taltobulin for 48 h in the presence or absence of 15 µM or 30 µM honokiol, 10 ng/mL FGF1 and 10 U/mL heparin. Cell viability was assessed using the PrestoBlue assay. Data were normalized to untreated cells. Statistical analysis was performed using an unpaired two-tailed t-test with GraphPad Prism 5. Data are shown as means ± SD from three independent experiments (n=3) with three replicates each. Statistical significance was defined as: ** $p \leq 0.01$, no significant differences denoted as ‘ns’.

This is a provisional file, not the final typeset article

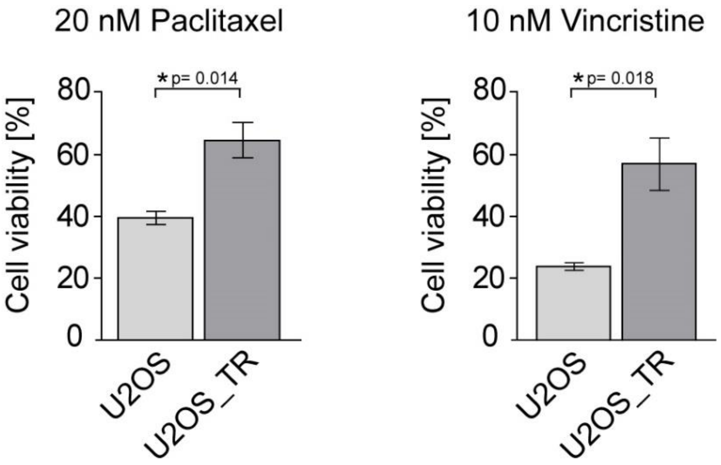

Supplementary figure 3. Evaluation of long-term exposure of U2OS cells to taltobulin on the development of chemoresistance to other tubulin-targeting drugs: paclitaxel and vincristine. Parental U2OS cells and derived U2OS_TR were treated with 20 nM paclitaxel or 10 nM vincristine for 48 h, and then their viability was assessed using the PrestoBlue assay. Data were normalized to untreated cells. Statistical analysis was performed using an unpaired two-tailed t-test with GraphPad Prism 5. Data are shown as means ± SEM from three independent experiments (n=3) with three replicates each. Statistical significance was defined as: * $p \leq 0.05$.
